# Postmortem 9.4-T MRI for Fetuses With Congenital Heart Defects Diagnosed in the First Trimester

**DOI:** 10.3389/fcvm.2021.764587

**Published:** 2022-01-27

**Authors:** Huirong Tang, Yan Zhang, Chenyan Dai, Tong Ru, Jie Li, Jieyu Chen, Bing Zhang, Kefeng Zhou, Pin Lv, Renyuan Liu, Qing Zhou, Mingming Zheng

**Affiliations:** ^1^Department of Obstetrics and Gynecology, The Affiliated Drum and Tower Hospital of Medical School of Nanjing University, Nanjing, China; ^2^Department of Pathology, The Affiliated Drum and Tower Hospital of Medical School of Nanjing University, Nanjing, China; ^3^Department of Radiology, The Affiliated Drum and Tower Hospital of Medical School of Nanjing University, Nanjing, China; ^4^Department of Cardiac Surgery, The Affiliated Drum and Tower Hospital of Medical School of Nanjing University, Nanjing, China

**Keywords:** congenital heart defects, postmortem magnetic resonance imaging, first- trimester ultrasound scan, 94-T magnetic resonance images, prenatal ultrasound

## Abstract

**Objective:**

To evaluate the feasibility of 9. 4-T postmortem MRI (pm-MRI) for assessment of major congenital heart defects (CHD) cases terminated in the early stage of gestation.

**Methods:**

Fetuses with CHD detected by the detailed first-trimester ultrasound scan and terminated before 18 gestational weeks were recruited between January 2018 and June 2020. All fetuses were offered 9.4-T pm-MRI examinations and those terminated over 13^+6^ weeks were offered conventional autopsies simultaneously. Findings of pm-MRI were compared with those of conventional autopsy and prenatal ultrasound.

**Results:**

A total of 19 fetuses with major CHD were analyzed, including 6 cases of the atrioventricular septal defect, 5 cases of Tetralogy of Fallot, 3 cases of hypoplastic left heart syndrome, 1 case of tricuspid atresia, 1 case of transposition of the great arteries, 1 case of severe tricuspid regurgitation, and 2 cases of complex CHD. Pm-MRI had concordant findings in 73.7% (14/19) cases, discordant findings in 15.8% (3/19) cases, and additional findings in 10.5% (2/19) cases when compared with prenatal ultrasound. Pm-MRI findings were concordant with autopsy in all 8 CHD cases terminated over 13^+6^ weeks.

**Conclusion:**

It is feasible to exhibit the structure of fetal heart terminated in the first trimester clearly on 9.4-T pm-MRI with an optimized scanning protocol. High-field pm-MRI could provide medical imaging information of CHD for those terminated in the early stage of gestation, especially for those limited by conventional autopsy.

## Introduction

Congenital heart defects (CHDs), which occur with an incidence of about 5–9 per 1,000 live births, are the most common malformations and the leading causes of neonatal death (1). Ultrasonography, especially echocardiography, is the mainstay for the diagnosis of CHDs prenatally. It is a safe and highly sensitive test that can be employed typically at the second trimester of pregnancy (2). With the development of modern high-resolution ultrasound techniques, a great amount of major CHDs including atrioventricular septal defects (AVSDs), transposition of the great arteries, tetralogy of Fallot, hypoplastic left heart, and other complex defects were reported in the first trimester of pregnancy (3, 4). The diagnosis of fetal CHDs in the first trimester may give health providers and parents the possibility of scheduling prenatal care or choosing to terminate the pregnancy in cases with poor prognosis earlier (5), while the wide implementation of fetal cardiac screening in the first trimester has raised concerns recently. A major concern is the lack of postmortem methods to confirm, especially for those terminated in the early gestational weeks as a conventional autopsy is not always possible considering the small size of the fetal heart (6, 7). Even stereomicroscopic autopsy had a 28.1% failure rate in the first trimester in the previous research (8).

Imaging techniques such as MRI, ultrasound, and CT as a part of postmortem examinations have been widely studied (9–11). Postmortem MRI (pm-MRI) using 1.5/3.0-T magnets offers an overall diagnostic accuracy of 77–94% and is probably one of the best choices as a virtual autopsy technique for fetuses terminated over 20 weeks of gestation (12, 13). However, the resolution of conventional pm-MRI at 1.5/3.0 T for small fetal organs, particularly the fetal heart, remains unsatisfied (14, 15). Recent studies showed high-field (4.7-/7.0-/9.4-T) MRI was a feasible option for postmortem examination of small fetuses and could provide good tissue characterization (8, 14, 16). While limited studies investigated the value of high-field pm-MRI in the assessment of CHDs detected and terminated in the first trimester (8, 17).

Thus, this study aimed to explore the use of 9.4-T pm-MRI in the reassessment of major CHD fetuses detected and terminated in the early stage of pregnancy.

## Materials and Methods

### Study Participants and Design

This was a single-center study conducted at Nanjing Drum Tower Hospital. The fetuses were recruited based on an ongoing prospective cohort study, which focused on the performance of a detailed first-trimester ultrasound (FTU) scan in detecting fetal structural and chromosomal anomalies (18, 19).

The inclusion criteria were as follows: (1) singletons with abnormal cardiac findings detected in the first trimester between January 2018 and June 2020; (2) pregnancy was terminated before 18 gestational weeks; (3) parents agreed to undergo pm-MRI and conventional autopsy (available in fetuses over 13^+6^ gestational weeks).

This study was approved by the Local Ethics Committee (2013058) and all the parents signed written informed consent.

### Procedure

#### Prenatal Ultrasound Scan

The singletons with crown-rump length from 45 to 84 mm were performed nuchal translucency measurement and detailed FTU scan including basic cardiac structural examination at our institution according to a standardized protocol (19). All suspected cardiac defects cases were referred to the specialist who had obtained The Fetal Medicine Foundation Certificate of Competence in ultrasound examination for fetal echocardiography for echocardiography to reassess the diagnosis. Consecutive fetal heart sonographic video clips were recorded in Viewpoint 6.0 (GE Healthcare GmbH, Munich, Germany, UK). All the ultrasound examinations were performed on Voluson E8 (GE Healthcare Austria GmbH & Co OG, Tiefenbach, Austria) ultrasound equipment with transabdominal transducer (4–8 MHz) and transvaginal transducer (5–9 MHz). The maternal characteristics and medical history were also collected and recorded in Viewpoint 6.0 (GE Healthcare GmbH, Munich, Germany, UK).

#### Preparation Before pm-MRI

**Figure 1:**
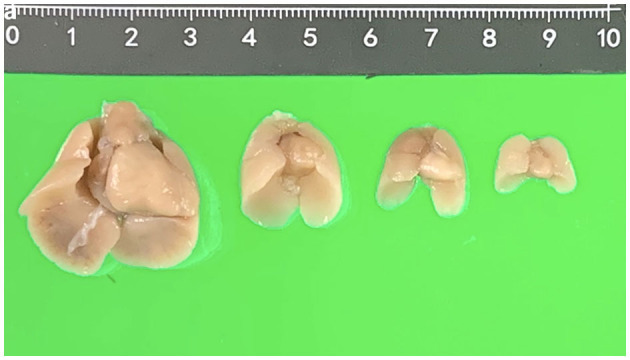
Fetal hearts and lungs in different gestational weeks (17, 15, 13, and 12 weeks, respectively).

After termination of pregnancy (TOP), one senior fetal pathologist (JY Chen) performed the initial external examination of the fetuses and took out the visceral organs including intact fetal heart and lungs from the thorax carefully. Then, the fetal visceral organs were fixed in 10% formalin solution with an optimum plastic tube (5, 15, 20, and 50 ml) according to the organ size and kept in the refrigerator at 4°C for more than 2 weeks before pm-MRI examination (Figure 1).

#### Postmortem MRI Examination

The MRI scan was carried out using a horizontal bore 9.4-T Bruker Biospec system (Biospec 94/20 USR, Bruker Biospin, Ettlingen, Germany, UK), equipped with the integrated Gradient and Shim Systems BGA12-S (Bruker Biospin) with a maximum magnetic field strength of 400 mT/m. Parameters were set according to different fetal gestational weeks, with a 0.3-mm slice thickness, an intersection gap of 0 mm, a field of view of 32 × 32 mm, matrix of 256 × 256 × (80–120) mm^3^, time of repeatation (TR)/time of echo (TE) of 6 ms/2.9 ms, averages 3, resulting voxel resolution of 0.100 × 0.100 mm^3^ (Table 1). Axial and coronal two-dimensional (2D) images were obtained to allow proper orientation of the subsequently obtained three-dimensional (3D)-T1-weighted images. On the 3D acquired volumes and whenever needed, regions of interest such as the chambers of the heart or the great vessels delineated slice-by-slice in each plane. Two MRI radiographers (P Lv and RY Liu) did all the pm-MRI scans.

**Table 1:** Typical MRI variables for three-dimensional T1-weighted sequence.

**Variables**	**Values**		
	**16^+0^-18^+0^ weeks**	**14^+0^-17^+6^ weeks**	**12–13^+6^weeks**
Slice thickness	0.3	0.3	0.3
Effective echo time (ms)	2.9	2.9	2.9
Relaxation time (ms)	6	6	6
Field of view (mm)	32 × 32 × 36	32 × 32 × 25	25 × 25 × 18
Matrix (data points)	256 × 256 × 120	256 × 256 × 80	250 × 250 × 60
Voxel size (mm)	0.125 × 0.125	0.125 × 0.125	0.100 × 0.100
Intersection gap (mm)	0	0	0
Scan time (min)	10–15	7–8	3–5
Flip angle (degree)	10	10	10
Number of signal average	3	3	3

#### Image Evaluation

Magnetic resonance imaging reports were made regarding the following regions of interest: the situs, abnormalities at the level of the four-chamber view, the left and right outflow tracts, the arch of the aorta, and the systemic veins. Pm-MRI interpretation was analyzed by a radiologist and a cardiothoracic surgeon together who had over 15 years of clinical experience. They were blinded to the ultrasound findings.

#### Conventional Pathologic Examination

After pm-MRI, visceral organs from fetuses terminated over 13^+6^ weeks were subjected to autopsies and microscopic examinations conducted by the same senior fetal pathologist. The pathologist was unaware of the results of the prenatal ultrasound examinations or those of the MRI examinations. The autopsy data were entered into a database that was separate from that used for the MRI examinations. At the end of the study, HR Tang who had access to all databases collated all data.

### Statistical Analysis

The Statistical Product and Service Solution (version 26.0 for Macintosh, SPSS, IBM Incorporation, Armonk, New York, USA) was used for data analysis. Continuous variables are reported as mean ± SD or median (range) depending on the data distribution. Categorical variables were expressed in percentages and frequencies.

## Results

A total of 82 (0.8%) fetuses were detected with abnormal cardiac findings in the first trimester from 9,688 consecutive single fetuses who underwent prenatal detailed FTU scan.

Except 6 cases continuing pregnancy, 22 cases terminated after 18 gestational weeks and 35 cases refusing to participate in the study, a total of 19 fetuses were finally analyzed, including 6 cases of AVSD, 5 cases of Tetralogy of Fallot, 3 cases of hypoplastic left heart syndrome, 1 case of tricuspid atresia, 1 case of transposition of the great arteries TGA, 1 case of severe tricuspid regurgitation, and 2 cases of complex CHD. The median gestational age of TOP was 13^+4^ weeks (12^+6^-18^+0^ weeks), including 4 cases between 12^+0^ and 13^+0^ weeks, 9 cases between 13^+1^ and 15^+0^ weeks, and 6 cases between 15^+1^ and 18^+0^ weeks. Except for 4 cases without genetic examination (refused to perform), 93.3% (14/15) cases had abnormal genetic results by chromosomal microarray (CMA) tests (7 cases of trisomy 18, 1 case of trisomy 21, 2 cases of trisomy 13, 2 cases of Turner syndrome, and 2 cases of 22q11 deletion) (Table 2).

**Table 2:** The prenatal diagnosis, postmortem MRI, and conventional autopsy findings in the 19 fetuses with abnormal cardiac findings.

**Case**	**Prenatal ultrasound findings**			**GA at TOP**	**Chromosomal results**	**Pm-MRI findings**	**Conventional** **autopsy findings**
	**Cardiac abnormality**	**Extracardiac** **abnormalities**	**GA at diagnosis**				
1	AVSD	Single umbilical artery	12+3	13+0	Normal	Malalignment VSD	Not performed
2	Complex CHD (AVSD, great vessels anomaly)	–	12+2	13+0	NA	AVSD TOF	Not performed
3	AVSD	Cystic hygroma and upper limb anomaly	12+0	13+3	Trisomy 18	AVSD	Not performed
4	TOF	Exomphalos	13+0	17+5	Deletion 22q11	TOF	TOF
5	AVSD	Exomphalos	13+2	13+5	Trisomy 13	AVSD	Not performed
6	TOF	Megalocystis	13+0	13+1	Trisomy 18	TOF	Not performed
7	TOF	–	12+6	13+1	NA	TOF	Not performed
8	TOF	–	12+4	15+0	Deletion 22q11	TOF	TOF
9	TOF	Increased NT	13+5	15+0	Trisomy 18	TOF	TOF
10	HLHS	Cystic hygroma	13+6	15+5	Monosomy X	HLHS	HLHS
11	HLHS	Increased NT	13+3	13+4	NA	HLHS	Not performed
12	Complex CHD (HLH, CoA?)	Increased NT	12+1	12+6	Monosomy X	CoA	Not performed
13	AVSD	Cystic hygroma, upper limb anomaly	13+0	16+2	Trisomy 18	AVSD	AVSD
14	HLHS	Holoprosencephaly, cleft palate, megalocystis	12+2	13+3	Trisomy 13	HLHS	Not performed
15	Severe tricuspid regurgitation	Cystic hygroma	13+1	17+6	Trisomy 21	AVSD	AVSD
16	Tricuspid atresia	–	12+2	16+3	NA	Tricuspid atresia	Tricuspid atresia
17	TGA	Increased NT, exomphalos, clench hands	13+4	17+6	Trisomy 18	DORV	DORV
18	AVSD	Radius anomaly and clench hands	12+3	13+0	Trisomy 18	AVSD	Not performed
19	AVSD	Cystic hygroma, clench hands, single umbilical artery	12+6	13+4	Trisomy 18	AVSD	Not performed

*GA, gestational age; TOP, termination of pregnancy; NA, not available; pm-MRI, postmortem magnetic resonance imaging; CHD, congenital heart disease; HLHS, hypoplastic left heart syndrome; AVSD, atrioventricular septal defect; TOF, tetralogy of fallot; HLH, hypoplastic left heart; CoA, coarctation of aorta; TGA, transposition of the great arteries; VSD, ventricular septal defect; DORV, double outlet right ventricle*.

### Evaluation of Cardiac Anatomy

**Figure 2:**
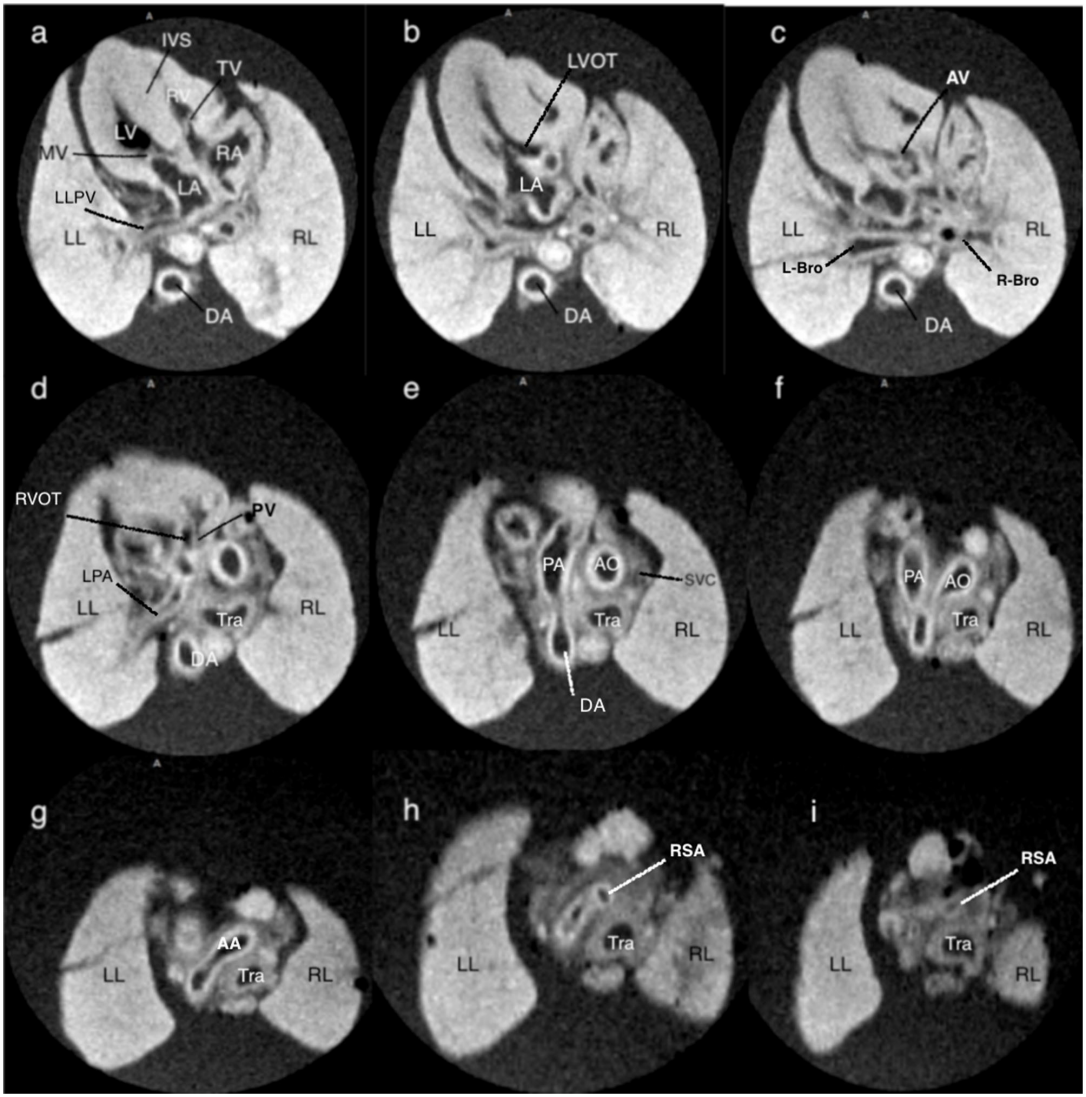
T1-weighted magnetic resonance consecutive images at 9.4 T of a fetus with normal cardiac anatomy, following termination of pregnancy at 13+1 weeks of gestation. **(a)** at the level of four-chamber view, showing a normal situs of the heart surrounded by the lungs, and a normal four-chamber view. **(b,c)** at the level of the left outflow tract showing the medial aortic wall was continuous with the ventricular septum and aortic valve. **(d)** at the level of the right outflow tract with a pulmonary artery that originates from the right ventricle with the clear bifurcation of the main pulmonary artery and left pulmonary artery. **(e)** at the level of the three-vessel view, the main pulmonary artery, ascending aorta, and superior vena cava are arranged in a straight line that extends from the left anterior to the right posterior. **(f)** at the level of the three-vessel and trachea view showing the transverse aortic arch and isthmus merge into the descending aorta, as does the pulmonary trunk and ductus arteriosus, creating a V-shaped configuration. **(g–i)** showing the right subclavian artery that branches off the aorta. LA, left atrium; LV, left ventricle; RA, right atrium; RV, right ventricle; RL, right lung; LL, left lung; TV, tricuspid valve; MV, mitral valve; LLPA, left lung pulmonary vein; DA, descending thoracic aorta; IVS, interventricular septum; LVOT, left ventricle outflow tract; AV, aortic valve; L-Bro, left bronchus; R-Bro, right bronchus; LPA, left pulmonary vein; RVOT, right ventricle outflow tract; Tra, trachea; PA, pulmonary artery; AO, aorta; AA, aortic arch; SVC, superior vena cava; RSA, right subclavian artery.

One fetus terminated for orofacial cleft without cardiac anomalies at 13+1 gestational weeks was recruited for MRI exploratory scan and its cardiac structure MRI was shown in Figure 2. It showed consecutive MRI of fetal cardiac structure (four-chamber view of atria and ventricles, crux and atrioventricular valves; three-vessel view of the pulmonary artery, aorta, and superior vena cava; other structures such as branches of pulmonary veins, left/right bronchus, and right subclavian artery). Video corresponding to these images is available as Movie I in the Data Supplement.

All the 19 cases underwent pm-MRI examinations successfully and 8 of 19 cases (42.1%) terminated over 13^+6^ weeks were subjected to autopsies.

**Figure 3:**
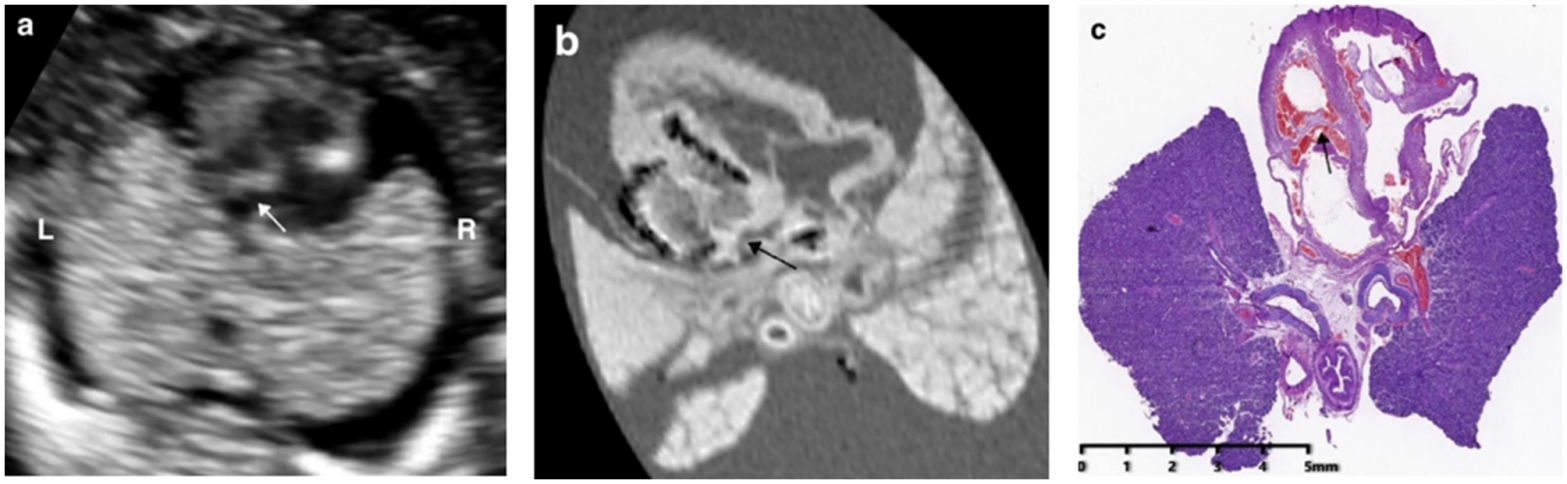
Prenatal ultrasound, postmortem MRI, and microscopic evaluation in a fetus with hypoplastic left heart syndrome terminated at 15+5 weeks of gestation, showing the left ventricle was smaller than the right ventricle significantly and the arrows showed mitral valve atresia.

In 73.7% (14/19) cases (case 3–11, 13–14, 16, 18, 19), the pm-MRI diagnosis was consistent with the prenatal ultrasound scan in the first trimester and a representative case of hypoplastic left heart syndrome was shown in Figure 3.

**Figure 4:**
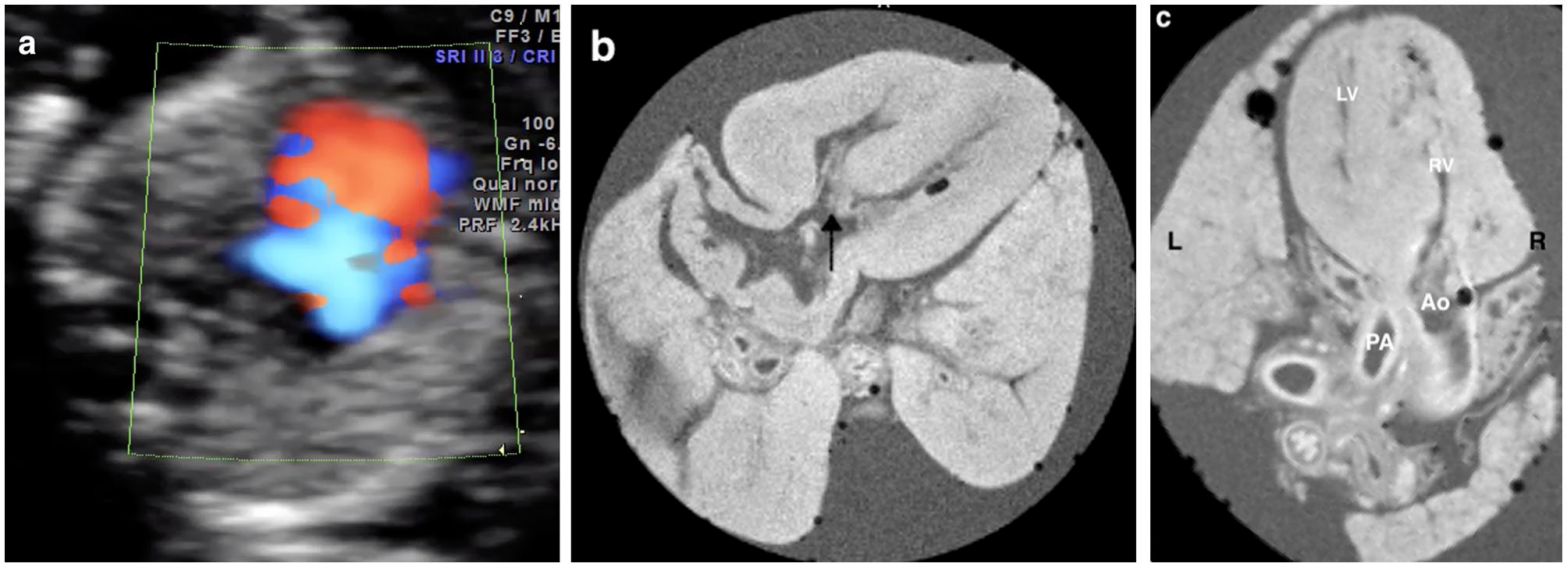
Discordant findings of postmortem MRI and autopsy compared with prenatal ultrasound. **(a)** A fetus presented with severe tricuspid regurgitation on the color Doppler imaging in the first trimester and was terminated at 17+6 weeks of gestation for Trisomy 21 (Case 15 in Table 2); **(b)** Four-chamber view showed the atrioventricular septal defect (AVSD) (black arrow) on the postmortem MRI; **(c)** Microscopic evaluation (H&E) assessed the AVSD.

**Figure 5:**
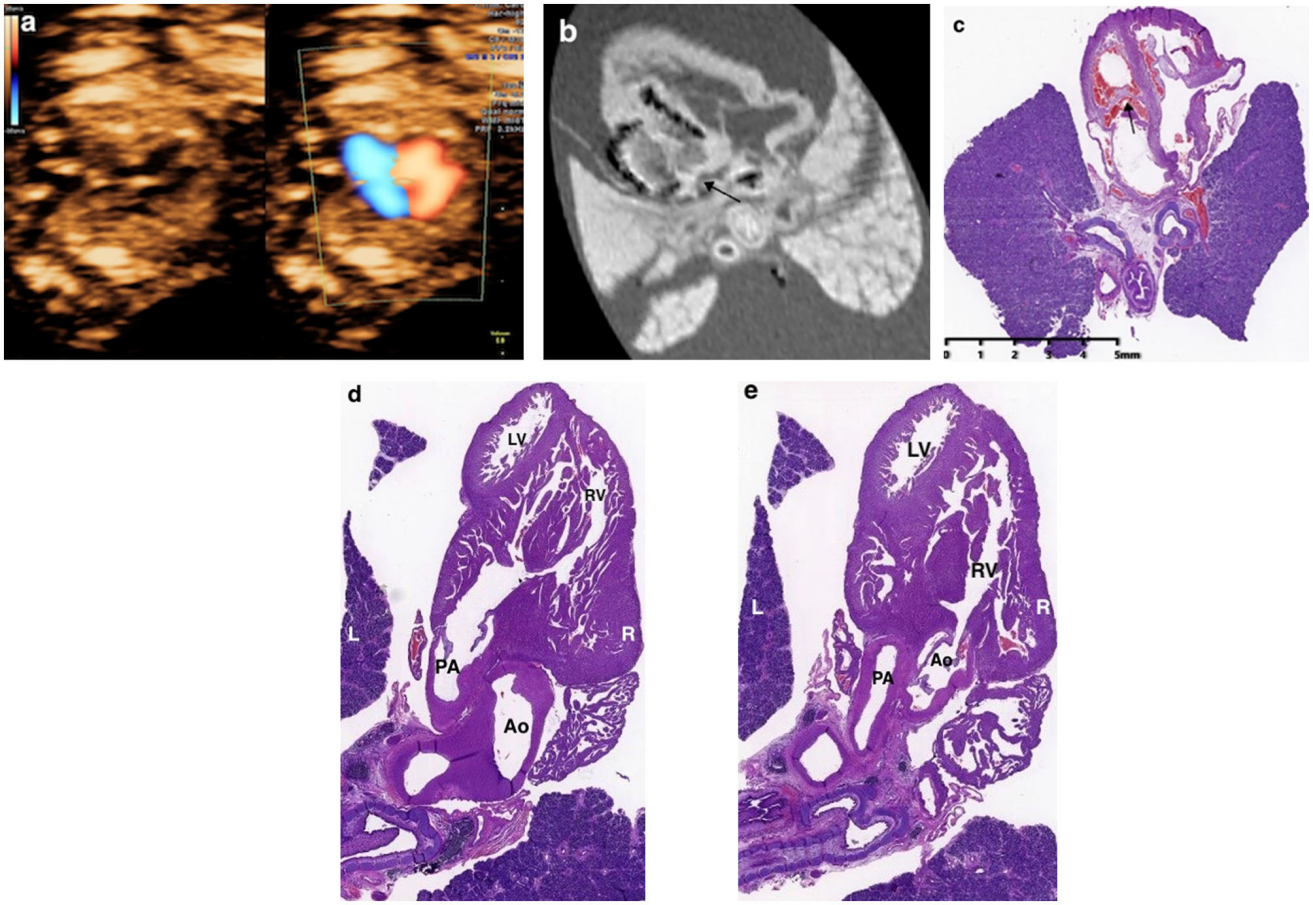
Discordant findings of postmortem MRI and autopsy compared with prenatal ultrasound. A fetus (Case 17 in Table 2) was detected as transposition of the great arteries **(a)** and was terminated at 17+6 weeks of gestation for Trisomy 18. The postmortem MRI showed both aortic artery and pulmonary artery arose entirely from the right ventricle with the aorta to the right and anterior to the pulmonary artery. **(b,c)** The autopsy confirmed the diagnosis of double-outlet right ventricle **(d,e)**.

**Figure 6:**
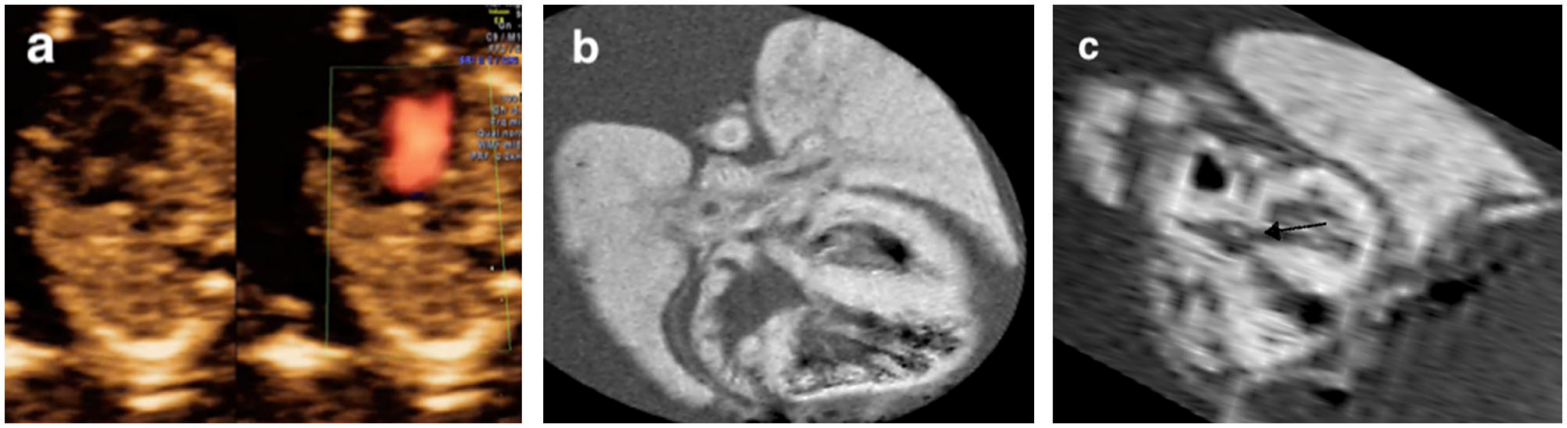
Discordant findings of postmortem MRI compared with prenatal ultrasound. **(a)** A fetus presented with an AVSD on the four-chamber view and was terminated at 13+0 gestational weeks (Case 1 in Table 2). Postmortem MRI showed intact atrioventricular septal **(b)** and left ventricle outflow tract showed malalignment ventricular septal defect (**c**, black arrow).

The pm-MRI findings were inconsistent with prenatal ultrasound scan in 15.8% (3/19) cases including 2 cases with autopsy assessment: (a) in case 15 (Table 2), the fetus was found severe tricuspid regurgitation but failed to yield a precise diagnosis prenatally. The fetus was terminated at 17 gestational weeks due to Trisomy 21 diagnosed by CVS. The pm-MRI revealed an AVSD clearly and the autopsy confirmed the diagnosis (Figure 4); (b) in Case 17 (Table 2), the fetus was detected as transposition of the great arteries in FTU scan and was terminated at 17^+6^ weeks of gestation for trisomy 18. The pm-MRI showed both the aortic artery and pulmonary artery completely arose from the right ventricle and the autopsy confirmed the diagnosis of double-outlet right ventricle (Figure 5). (c) in Case 1 (Table 2), pm-MRI examination of the heart revealed malalignment ventricular septal defect instead of AVSD detected by FTU (Figure 6).

**Figure 7:**
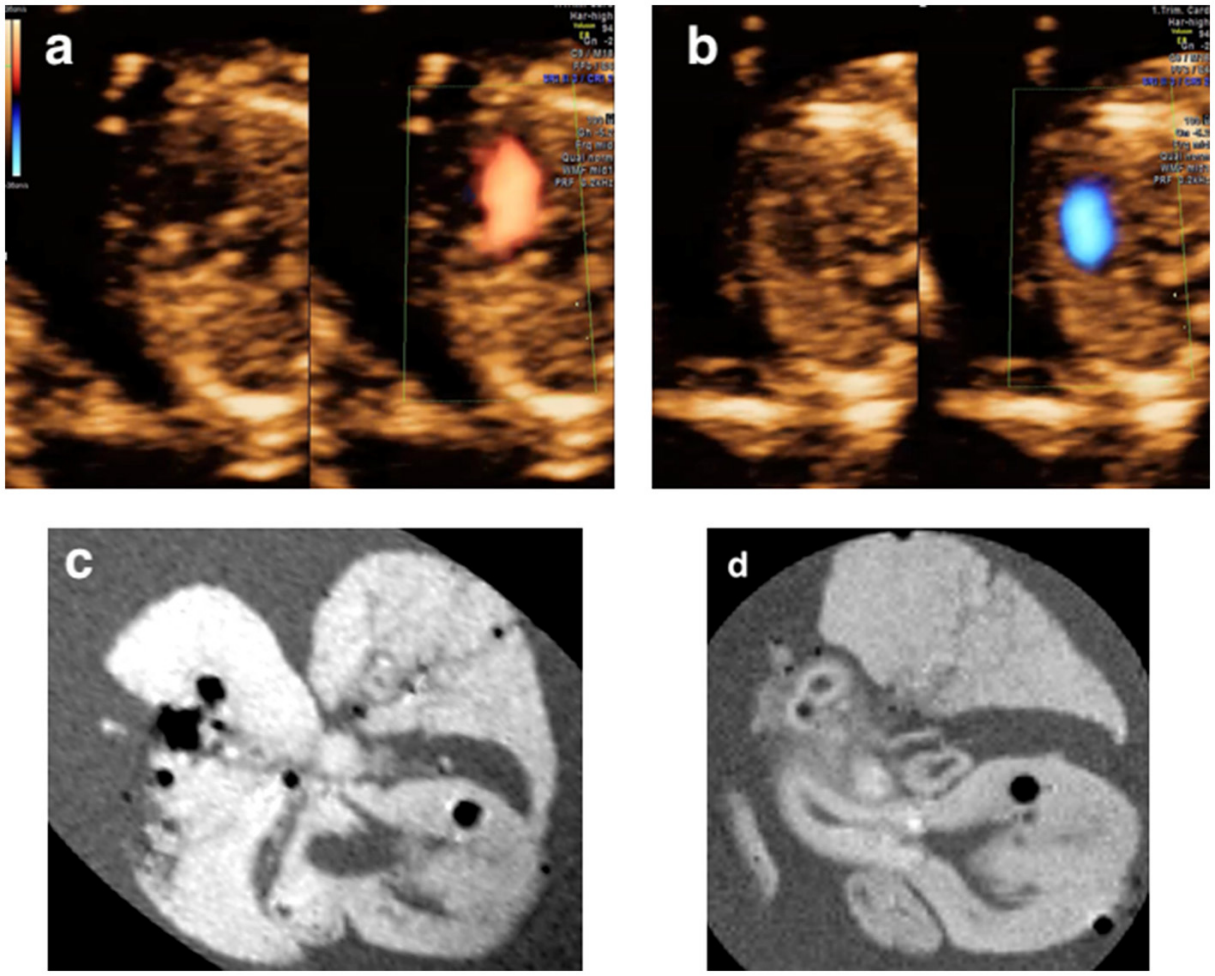
**(a,b)** A fetus terminated at 13+5 weeks of gestation and diagnosed as AVSD and suspected of great vessels malformation (Case 2 in Table 2); **(c,d)** Post-mortem MRI showing the AVSD at the four-chamber view and an overriding aorta at left ventricle outflow tract view.

**Figure 8:**
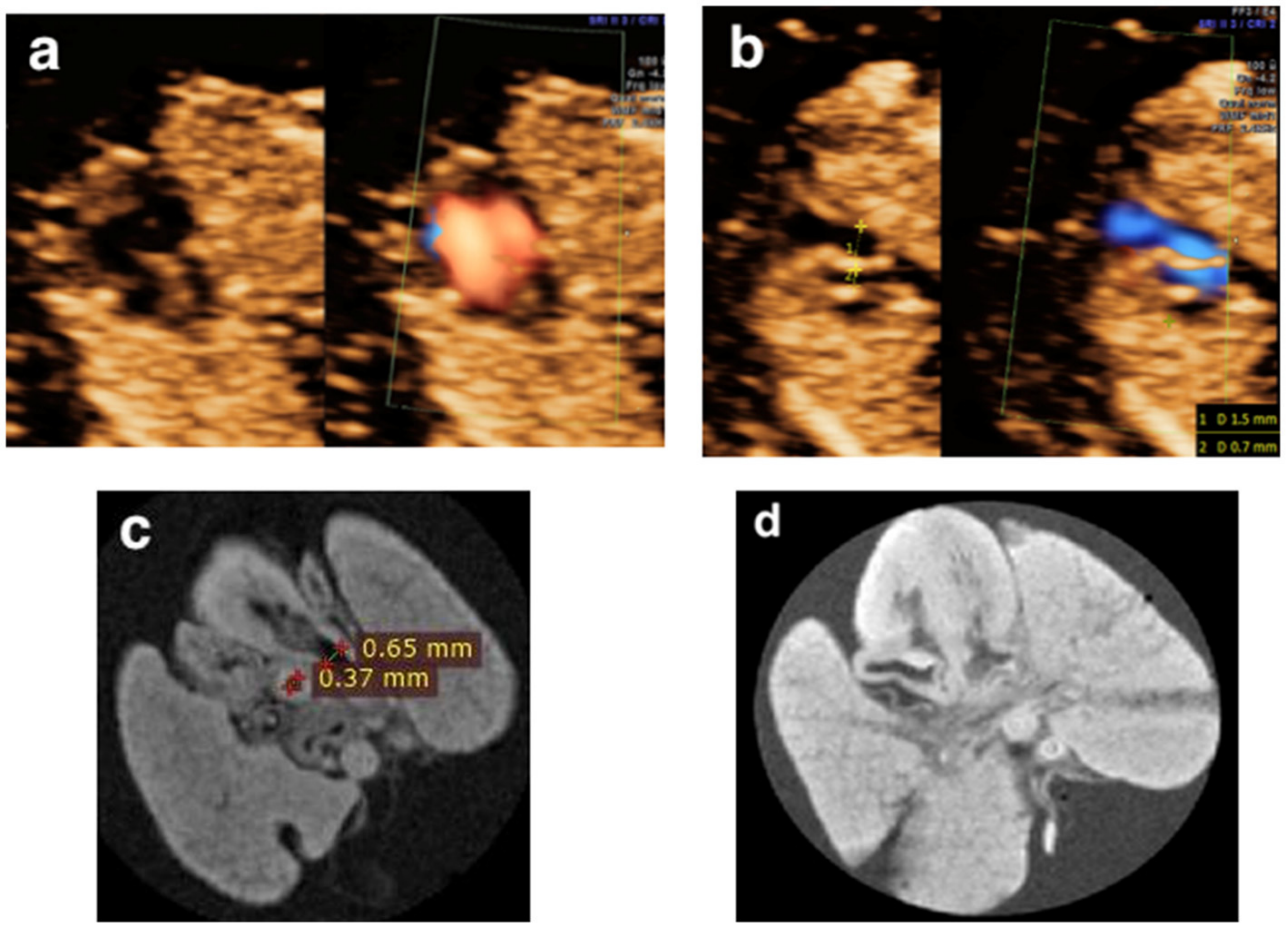
**(a,b)** A fetus presented with a smaller left ventricle on the four-chamber view and a larger pulmonary artery diameter than that of the aorta (1.5 vs. 0.7 mm) at the three-vessel view in the first trimester and was terminated at 12+6 weeks of gestation for Turner syndrome (Case 12 in Table 2). **(c,d)** Postmortem MRI showed a normally connected heart with the crossing of the great vessels and a larger pulmonary artery diameter than that of the aorta (0.65 vs. 0.37 mm).

In 10.5% (2/19) cases, pm-MRI provided additional information: (a) in Case 2 (Table 2), the fetus was diagnosed as AVSD and suspected of great vessels malformation prenatally. After TOP, AVSD, and Tetralogy of Fallot were observed using pm-MRI (Figure 7); (b) in Case 12 (Table 2), the hypoplastic left heart was observed and coarctation of aorta was suspected in a prenatal ultrasound scan. The fetus was terminated at 12 gestational weeks and was diagnosed with Turner syndrome by CMA postnatally. Pm-MRI showed that the aorta diameter was significantly smaller than the pulmonary artery (0.37 vs. 0.65 mm) on the three-vessel view and the left ventricle was smaller than the right ventricle on the four-chamber view (Figure 8). Thus, the diagnosis of aortic coarctation was raised by pm-MRI.

## Discussion

### Main Findings

This study showed that it was clear to visualize fetal heart structure in detail using 9.4-T pm-MRI with an optimized scanning protocol. Pm-MRI had concordant findings in 73.7% (14/19) cases, discordant findings in 15.8% (3/19) cases, and additional findings in 10.5% (2/19) cases when compared with prenatal ultrasound. Pm-MRI findings were concordant with autopsy in all the 8 CHD cases terminated over 13^+6^ weeks.

The implementation of a detailed FTU scan has fundamentally changed prenatal care by moving the detection of major structural abnormalities, including cardiac abnormalities, to the early stage of gestation. A study by Syngelaki A showed that FTU scan in 11–13^+6^ weeks could diagnose all cases of tricuspid or pulmonary atresia, more than 90% cases of hypoplastic left heart syndrome, and AVSD (4). Our previous prospective study also suggested the vast majority of major CHDs (83.3%) can be detected during detailed FTU performed by experienced sonographers (18, 19). However, there is a longstanding desire for a postmortem reassessment of ultrasound diagnosis, especially in certain forms of CHDs terminated in the early stage of gestation. This study focused on postmortem assessment of fetuses with CHD diagnosed in the first trimester. In this study, we established a cohort based on a detailed FTU scan, which enables us to investigate a variety of CHD cases detected in the first trimester. Also, we developed the optimized protocol for pm-MRI examination of fetal hearts with TOP ranging from 12^+6^ to 17^+6^ gestational weeks. Studies have reported different methods of pre-scanning preparation before pm-MRI. A study by Votino C showed that postmortem fetal tissues were frozen at −20°C before an MRI scan could be beneficial for cardiac imaging (12). A study by Staicu A indicated that using 10% formalin solution before MRI scanning is beneficial for MRI imaging (6). In order to further improve the resolution of pm-MRI, we took out the visceral organs including intact fetal heart and lungs and then fixed them within 10% formalin for more than 2 weeks before the MRI scan. Though this could compromise the integrity of the fetus, it seems to be beneficial for improving the resolution of pm-MRI with presenting precise cardiac anatomy because of the complete immersion with formalin (Figure 2). Furthermore, we conducted exploratory scans using three fetal hearts in different gestational weeks terminated for extracardiac anomalies only. To obtain an optimal resolution, we found setting different parameters according to different fetal sizes before 18 weeks was essential. Thus, we conducted the MRI scan for CHD fetuses in different parameters according to their gestational weeks within practicable scanning time (<20 min each case) (shown in Table 1).

The diagnosis of CHD may be missed or wrongly interpreted in the antenatal scan, especially in the early gestational stage (20, 21). Zidere and colleagues demonstrated that their expertise in first-trimester diagnosis in continuing pregnancies scanned at least 6 weeks later had a false-positive diagnosis of CHD in 7/81 (9%) cases (22). Therefore, it was crucial to perform a postmortem evaluation of those terminated for CHD in the early stage. This study reported a false-positive case with AVSD diagnosis prenatally and pm-MRI revealed features of malalignment VSD. On the other hand, around 10% of fetuses terminated for aneuploidies below 14 weeks were non-diagnostic structural anomalies on the prenatal ultrasound (23). This study showed that 9.4-T pm-MRI could further reveal precise CHD diagnosis (AVSD) with clear images in the fetus terminated for Trisomy 21 with only tricuspid regurgitation on FTU (Case 15 in Table 2). The comparatively ideal performance of high-field pm-MRI for small fetal hearts in this study is encouraging, especially for those who were unavailable for conventional autopsy due to size restrictions.

In addition, it is impossible to always give a precise diagnosis of CHD in the early stage of pregnancy by prenatal ultrasound (24). For those suspicious of severe CHD without a precise diagnosis prenatally, the results of this study indicated that 9.4-T pm-MRI could provide the sound morphological basis for assessment of cardiac malformation terminated in the early stage. In Cases 2 and 12 (Table 2), more diagnostic information was provided by pm-MRI, and, therefore, a supplementation to the diagnosis was made.

### Study Limitations

Pathologic results of 11 cases in this study were not available owing to the technical limitations of the postmortem examination though pm-MRI provided us clear images of the cardiac structure of these cases. Considering the study is confined to a single center and the limited number of CHD cases, it requires more validation studies in more centers.

## Conclusion

This study showed that it is feasible to exhibit fetal cardiac structure terminated in the early stage of gestation clearly on 9.4-T pm-MRI with an optimized scanning protocol. High-field pm-MRI could provide imaging information for CHD fetuses, especially for those terminated in the early stage of pregnancy limited by conventional autopsy.

## Data Availability Statement

The original contributions presented in the study are included in the article/Supplementary Materials, further inquiries can be directed to the corresponding author/s.

## Ethics Statement

The studies involving human participants were reviewed and approved by Ethics Committee of the Affiliated Drum Tower Hospital of Medical School of Nanjing University. The Reg No. is 2013058. The patients/participants provided their written informed consent to participate in this study. Written informed consent was obtained from the individual(s) for the publication of any potentially identifiable images or data included in this article.

## Author Contributions

MZ and HT designed the study, collected the data, performed the analyses, interpreted the results, and drafted the manuscript. MZ, YZ, CD, and TR performed the detailed first-trimester ultrasound in this study. JL performed the genetic tests in this study. JC performed the conventional autopsy. KZ and QZ interpreted the MRI results. MZ and YZ supervised the study design, data collection, analysis and interpretation, and reviewed and revised the manuscript. All authors contributed to the article and approved the submitted version.

## Funding

This study was supported by grants from the Six Talent Peaks Project in Jiangsu Province (WSN-141), Nanjing Youth Medical Innovation Personnel Training Project (QRX17014). The funding agencies did not have any role in the design of the study, the collection, analysis, and interpretation of data, and in writing the manuscript.

## Conflict of Interest

The authors declare that the research was conducted in the absence of any commercial or financial relationships that could be construed as a potential conflict of interest.

## Publisher's Note

All claims expressed in this article are solely those of the authors and do not necessarily represent those of their affiliated organizations, or those of the publisher, the editors and the reviewers. Any product that may be evaluated in this article, or claim that may be made by its manufacturer, is not guaranteed or endorsed by the publisher.

## Supplementary Material

Supplementary Video 1 The consecutive fetal heart video clips using postmortem MRI in a fetus without congenital heart defects.

Supplementary Video 2 The consecutive fetal heart video clips using postmortem MRI in Case 17 in Table 2.

Supplementary Video 3 The video clips of fetal echocardiogram of case 11 showing hypoplastic left heart syndrome in Table 2.

Supplementary Video 4 The video clips of fetal echocardiogram of case 17 showing transposition of the great arteries in Table 2.

Supplementary Video 5 The video clips of fetal echocardiogram of case 4 showing Tetralogy of Fallot in Table 2.

Supplementary Video 6 The video clips of fetal echocardiogram of case 5 showing atrioventricular septal defect in Table 2.
