# Supplementary material for: Postmortem 9.4-T MRI for Fetuses With Congenital Heart Defects Diagnosed in the First Trimester
Source: Front Cardiovasc Med. 2022 Jan 27;8:764587. doi: 10.3389/fcvm.2021.764587 (PMC8830519; doi:10.3389/fcvm.2021.764587)
Supplement: Supplementary file 1 [file Data_Sheet_1.docx]

The captions of Supplementary Videos 2–6

Supplementary video 2: The consecutive fetal heart video clips using postmortem MRI in Case 17 in Table 2.

Supplementary video 3: The video clips of fetal echocardiogram of case 11 showing hypoplastic left heart syndrome in Table 2.

Supplementary video 4: The video clips of fetal echocardiogram of case 17 showing transposition of the great arteries in Table 2.

Supplementary video 5: The video clips of fetal echocardiogram of case 4 showing Tetralogy of Fallot in Table 2.

Supplementary video 6: The video clips of fetal echocardiogram of case 5 showing atrioventricular septal defect in Table 2.
